# To Our Readers

**Published:** 1886-10-02

**Authors:** 


### TO OUR READERS.

"Be noble! and the nobleness that lies
In other men, sleeping, but never dead,
Will rise in majesty to meet thine own."

LOWELL.

Be noble! SUCH might well be the spirit, and such is no doubt largely the spirit, in which the many workers in our hospitals, asylums, and other institutions for the cure and relief of the sick and suffering, go about their several duties. For the institutions themselves, and for the managers also, no better spirit could be desired or imagined. To soothe the sufferers, to restore the injured, to comfort the dying, and to cure the sick must at all times be a God-like work, and one which cannot fail to ennoble the noblest work of God. If this be true, then the wider such influences are extended, the better for the nation and the people at large. And between the parish or congregation, the family or household, and even the individual and the hospital and its workers, there is that touch of nature, that golden chord of human sympathy, which makes the whole world kin. Every parish, congregation, family, household, and individual must encounter the dark days of human weakness, of which disease is the infallible magnet, as all must one day realise in their own persons, or in the persons of those who are nearest and dearest to them.

Our work. This being so, it will be the mission of THE HOSPITAL to aid all to provide against this day of trial, to face it happily, and to fight disease with the best weapons it can make available, by extending the area of public interest and knowledge in the work of those who labour amongst sickness and suffering. The public conscience is undoubtedly asleep to this now. It is because we believe, with Mr. Lowell, in "the nobleness that lies in other men, sleeping, but never dead," that we have undertaken to strive to bring under the notice of the people and the Churches, the nobility of the work of the hospitals, asylums, and kindred institutions, and of those who labour in them. May the men and women who slumber and sleep to-day, have "the nobleness of their natures aroused in majesty" to meet this great humanising influence, which it will be our mission to diffuse amongst churches and people !

A Good Cause. On the highest grounds, then, there is cause to nerve our energies to the task. On similar grounds and on low grounds, too—namely, those of self-interest,—there is cause enough why every clergyman and minister of religion, every head of a family or household, every man and woman of intelligence and good feeling, should stretch out the right hand of fellowship and join hands with us to make the effort as far-reaching, useful, and helpful as it undoubtedly ought to become. This is an age when self-reliance and self-help are recognised as the principles best calculated to promote individual happiness and success. Who shall estimate the sum of preventable human misery which at present prevails, and which is largely caused by a want of knowledge and co-operation amongst those who suffer or who have to provide for the sufferers, and those agencies and individuals which can give relief ? If this be indeed so,—and who will controvert it ?—then The Hospital ought to be gladly welcomed by every family and congregation as well as by the institutions, the work of which, and the wherewithal to do that work, will form the substance of much that we shall have to treat of.

Mothers and Ministers. How many a mother, how often a minister of religion, desires to know what to do till the doctor comes, or where to find out how to secure for a poor sufferer the particular form of relief which the sufferer needs to help him on his way ? Henceforth no such doubts need exist among the readers of The Hospital, and it is this knowledge which makes us believe that we shall be able to join hands with, and to help forward the classes, whom we wish to benefit.

Our Policy. What, then, is to be the policy of this paper? It will strive to provide entertaining, instructive, and amusing reading of a popular kind, which will be gathered from the hospital field, using the word hospital in its widest sense, and making it to include all agencies of whatever kind which have for their object the treatment and cure of disease, or the care of the sick and injured all the world over. There will be a serial story to commence with, founded upon some aspect of this side of life, and written by well-known authors. There will be the experiences and recollections of the many who have had to do with these various institutions as patients, officers, or medical attendants, together with the opinions of all the most eminent authorities on current topics of interest. The Hospital will contain columns for the curious, for historical recollections, for hospital worthies, for the children, for biographical and personal intelligence, and for notes and news, in addition to a madman's column, and information as to where to go to seek relief, together with a list of the leading members of the medical profession in every branch. A place will be found for sick-room requisites and cookery, nursing notes, the patients' hospital diary, district and private nursing, and a column in which information can be sought and found as to the treatment and disposal of difficult cases. To these will be added free employment columns, in which any mistress of a household, or lady superintendent, or matron, will be able to advertise for a certificated nurse or for a capable nursemaid on conditions which will be found in full on another page.

Of interest to all. An attempt will be made to further the wishes of those who desire to find employment in the hospitals, or to become members of the medical or nursing profession ; to aid the clergy, district visitors, the patients and their friends, to find employment for the convalescents on their discharge, with the view of preventing for the future such miseries and temptations as George Fleming so graphically describes in his story "Discharged," the first part of which appears in our present issue. The space devoted to ward and window gardening, ferns and artificial flowers and ward decorations, should cause the country to unite with the city in a combined and successful effort to lighten the hours of suffering and to brighten the surroundings of the sick. The Good Samaritan cases should aid the clergy and district visitors to find the help and assistance they need at times for the really deserving, whilst the chaplain's column should form a bond of sympathy between those who minister to parishes and congregations, and those whose main work lies with disease and death. The latent energies and talents of all will be appealed to by our system of Prize Competitions, whilst a column for questions will be started, in which information will be given to the many who at present are often sore beset at times, by unexpected difficulties at home, in the parish or congregation, and individually.

Co-operation invited. There are many other points upon which we might dwell, but we have said enough to show what we are about to attempt in the interests of vast numbers of the people and of all the institutions, and we confidently invite the co-operation of both sexes and all classes in this humanising work and labour of love. If we may judge by the success which attended the exertions in behalf of all the hospitals in connection with the three Special Supplements of the *Lancet*, recently published, then there can be no doubt that The Hospital will soon prove as popular, as it ought to be widely useful, to all sorts and conditions of men and women.

**The Hospitals and all kindred Institutions.** But apart from the people at large, that great aggregate of human souls which constitutes the English-speaking population of the civilised world, what does The Hospital wish to do for the hospitals, asylums, and kindred institutions, and for all within their walls? To the hospitals especially we desire to extend the strongest support, because we are painfully impressed with the necessity there is that more funds should be provided for this work. This absence of ready money is due to the non-existence of an extended area of public interest in the work which the hospitals do for the whole community, and to the consequent milking of the same cow year after year by nearly all the secretaries. What wonder if the cow shows every year increasing symptoms of being dry, or if the supply of milk by gradual diminution threatens discontinuance altogether for a time, if not for aye! The area of public interest must, therefore, be extended, and when the area of support is thus increased, adequate funds may be reasonably expected to follow. More cash and less criticism will, therefore, be the principle upon which this section of The Hospital will be conducted. Indeed, it has been started with the main object of largely increasing the support already given to hospitals, by awakening a personal and sustained interest in the work which they undoubtedly do for the whole community, and for each individual, from the highest to the lowest.

**Two Evils.** Two evils we shall steadily endeavour to combat. One, the vexatious, uncalled-for, often valueless, and always prejudiced, criticism of the persons whom the Americans call "Cranks" having an axe to grind. The other, the unnecessary multiplication of so-called charities, which are too often the outcome of personal considerations rather than of a regard for the public weal. We propose to tell the story of all the hospitals in these columns; and with the object of helping the managers of particular charities, we have made arrangements, at their request, for the publication of Special Supplements, which will be devoted entirely to the work, claims, and objects of individual institutions, the whole of which will be written in a popular form by experienced special correspondents. We shall at all times be ready to help all or any of the institutions which labour within the field covered by this paper. We invite a statement of the trials and difficulties, as well as of the needs, of particular charities, to which we shall always be prepared to give sympathetic consideration. We shall welcome the co-operation of any who have it in their power to help in this effort to promote the welfare of all institutions or agencies for the relief of sickness and disease, with their attendant evils.

**A Friend to Officials.** Finally, we desire to be the firm friend and supporter of all who labour within the walls of these institutions. The interests of the officials and of the patients, as well as those of the institutions and their managers, will be our interests also. Anything which interests them will interest us; and whilst we desire to make The Hospital a family, congregational, and household Journal, we shall never lose sight of the army of workers upon whose devoted labours the efficiency and success of these institutions must always mainly depend.

**The Prize Competitions.** The prizes offered from time to time will prove, we hope, interesting and helpful to the public, the patients, and to hospital and asylum officials, and we cordially acknowledge the warm welcome and the hearty assistance already extended to The Hospital by many of the authorities, and especially by representative secretaries, lady superintendents, matrons, sisters and nurses connected with the metropolitan and provincial, Scotch and Irish hospitals, asylums and kindred institutions.

**A Family and Institution Journal.** The Hospital will be a family newspaper devoted to their interests and to those of the Institutions. For this reason, and because we shall have no other object but to promote the welfare of families and congregations, the care of the lonely in sickness, and the adequate support and efficient management of all the charities, we confidently anticipate the hearty co-operation of heads of households, clergy and ministers, and all managers, officials and patients, from the highest to the lowest. If our anticipation prove to be well founded, Mr. Lowell's aspirations will have borne good fruit, and the nobleness that lies in other men, sleeping, but never dead, will indeed have risen in majesty to the benefit of our generation.

